# PTX3 orchestrates tissue repair

**DOI:** 10.18632/oncotarget.5453

**Published:** 2015-09-02

**Authors:** Andrea Doni, Cecilia Garlanda, Alberto Mantovani

**Affiliations:** Humanitas Clinical and Research Center and Humanitas University, Rozzano, Milan, Italy

**Keywords:** innate immunity, tissue repair

Innate immunity is the first line of resistance against pathogens, but it has also an important role in maintaining tissue homeostasis by regulating inflammatory and repair responses after tissue injury [1]. Innate immunity consists of a cellular and humoral arm [1, 2]. It has been clearly established that cell-associated pattern-recognition molecules (PRM) sense damage-associated molecular patterns [e.g. extracellular matrix (ECM) components, nuclear proteins and nucleic acids] released in injured tissues and initiate tissue repair process [1], whereas the role of humoral innate immunity molecules in tissue repair has been the object of limited attention. Recently, we have shown that the long pentraxin 3 (PTX3), an essential component of the humoral innate immunity [2], plays a non-redundant role in tissue repair and remodelling [3].

**Figure 1:**
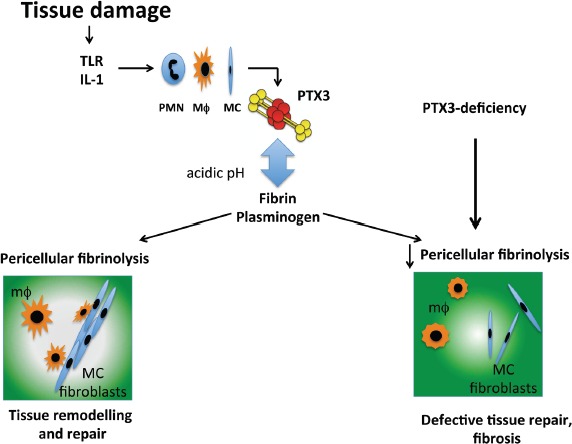
Schematic view of the role of PTX3 in tissue repair After injury, PTX3 produced by neutrophils (PMN), macrophages (MΦ) and mesenchymal cells (MC), interacts with fibrin matrix and plasminogen sustaining pericellular fibrinolysis by tissue remodelling cells and contributing to an appropriate tissue repair. The acidic pH, which occurs in the first phases of the tissue repair process, governs the interaction between PTX3 and fibrin and plasminogen. PTX3-deficiency is associated with impaired pericellular fibrinolysis and collective migration of remodelling cells, followed by increased fibrosis and defective tissue repair.

PTX3, the first member of the long pentraxin subfamily identified, is a highly conserved octameric glycoprotein constituted by a C-terminal pentraxin domain, homologous to the classic short pentraxins C-reactive protein (CRP) and serum amyloid P component (SAP), and an unrelated N-terminal domain. PTX3 is produced by parenchymal and immune cells in response to proinflammatory stimuli [2], has opsonic activity via Fcγ receptors, activates and regulates the complement cascade and regulates inflammation [2]. In an effort to define the functions of PTX3 in tissue damage, we observed in different models of tissue damage (skin wound healing, chemically-induced sterile liver and lung injury, arterial thrombosis) that PTX3-deficiency was associated with increased clot formation, fibrin deposition and persistence, followed by increased collagen deposition. *In vitro* and *in vivo* studies demonstrated that by interacting with fibrin and plasminogen (Plg) at acidic conditions, which occur in damaged tissues, PTX3 promoted remodelling of the fibrin-rich inflammatory matrix ensuring a normal tissue repair [3] (Figure 1).

In damaged skin, PTX3 was associated to the epithelial outgrowth and to bundles of aligned macrophages and mesenchymal cells that collectively invaded the wound site forming paths in the provisional fibrin matrix. Upon skin wounding, PTX3 was produced by macrophages and mesenchymal cells downstream of TLR sensing and IL-1 amplification, and once released, it localized in the pericellular matrix of remodelling cells. PTX3-deficient macrophages and mesenchymal cells failed to collectively migrate and form patent passages within fibrin matrix *in vivo*, and showed defective pericellular fibrinolysis *in vitro*, suggesting the importance of PTX3 in promoting the directional migration and invasive phenotype of remodelling cells within fibrin. Excessive fibrin accumulation and subsequent increased collagen deposition were also prominent anomalies observed in liver and lung repair.

These findings suggested the interaction of PTX3 with components of the haemostatic system and fibrinolytic cascade, since fibrin has a prominent role as a provisional matrix protein guiding subsequent repair [4]. Indeed, PTX3 was found to interact with fibrinogen (FG)/fibrin and Plg at acidic pH and to facilitate fibrin dissolution. The second exon-encoded N-terminal domain of PTX3 was responsible for the interaction with fibrin and Plg, which occurred through different sites. PTX3 did not interfere with the interaction between Plg and fibrin; indeed, in the presence of PTX3 the interaction between Plg and fibrin was amplified, indicating that PTX3 acted as a scaffold and a tripartite interaction among PTX3, fibrin and Plg at low pH promoted fibrin degradation. This activity is peculiar for PTX3, since in the same setting CRP did not interact with FG/fibrin or Plg. The antifibrotic potential of SAP has been attributed to the inhibition of monocyte differentiation to fibrocytes and to the activation of DC-SIGN leading to IL-10 production [5]. Moreover, *in vivo* administration of the PTX3 N-terminal domain, but not of the C-terminal pentraxin domain, was sufficient to revert the phenotype of PTX3-deficient mice in skin wound healing. The rescue of the phenotype of PTX3- deficient mice was also obtained by *in vivo* administration of selective inhibitors of fibrin deposition and platelet activation, thus demonstrating that an altered fibrinolytic response to injury was responsible for the defective wound healing.

Confocal and two-photon microscopy experiments using a pH sensitive fluorescent probe and fluorophore-conjugated specific antibodies demonstrated that the interaction between PTX3 and fibrin or Plg preferentially occurred *in vivo* in more acidic areas of the wound site. The acidification of the wound site consequent to the cell metabolic adaptation induced by tissue hypoperfusion and hypoxia, is a key determinant for this interaction. This ensures that the tripartite interaction does not occur in the circulation but rather at sites of tissue repair.

Thus, a molecule belonging to the humoral arm of innate immunity is involved in tissue repair by interacting with components of the provisional ECM and promoting fibrin remodelling. An acidic pH sets PTX3 in a tissue repair mode. These findings provide a novel link between inflammation, immunity, haemostasis and tissue repair [6]. Other humoral PRMs, such as C1q, collectins, CRP and SAP, interact with ECM components, and several ECM components such as fibronectin, mindin, osteopontin, and vitronectin are involved in microbial recognition. The role of fibrinogen domain-containing proteins in invertebrates is bacterial recognition, agglutination and lysis, but not coagulation, subsequently acquired in vertebrates [7], and in vertebrates, several fluid-phase PRM contain ancestral collagen and fibrinogen domains [2]. The present study is in line with these lines of evidence and point to the evolutionary link between microbial and ECM recognition in the humoral arm of innate immunity.
